# Co-Operation of City Corporation and Freemen

**Published:** 1917-05-19

**Authors:** 


### ROYAL VICTORIA INFIRMARY, NEWCASTLE.

### Co-operation of City Corporation and Freemen.

EXTENSIVE additions are to be made to the Royal Victoria Infirmary, Newcastle-on-Tyne. These extensions are due entirely to the local needs of a huge industrial population—shipbuilding, coal-mining, munition works, iron foundries, etc.—to meet which the hospital accommodation is at present totally inadequate.

The Newcastle Corporation and the Guild of Freemen have agreed unanimously to grant an application made by the House Committee of the infirmary, on behalf of the governors, for a gift of 14¾ acres of land known as the Leazes, adjoining the present large and modern building. This makes a total of nearly 25 acres handed over by the local authorities for the building of extensions of the institution. All the land is public land vested in the Newcastle Corporation, and the Freemen hold the grazing rights.

It is pleasant to be able to record that both the Corporation of Newcastle and the Freemen met the infirmary authorities more than half-way, and are only too anxious to help. The Freemen have surrendered their Herbage Rights as a free gift. The passing of the Bill in Parliament and the transfer of the whole business will cost not more than a flea-bite, which the infirmary authorities are only too glad to bear.

The provision of additional accommodation has been very urgent for over a year now. In the present building prior to the outbreak of war there were 430 beds. Even with this number there were large numbers on the waiting lists. When war broke out 200 beds were devoted to military patients, and to meet this situation temporary wards were erected giving accommodation for fifty-two beds. By utilising the out-patients' hall and providing other beds throughout the hospital the total number of beds was increased to 567, leaving available for the accommodation of civil patients 367 beds. Owing to the great influx of workers to the shipyards and factories in the neighbourhood, the number of accident cases increased, with the result that the whole of the civil cases waiting for treatment could not be dealt with. There were over 2,000 cases on the waiting-list, of which 1,500 were surgical cases awaiting operations.

The Infirmary Committee also undertook to provide accommodation for the treatment of patients suffering from venereal diseases in Newcastle and the counties of Northumberland and Durham. At considerable trouble small temporary accommodation was found for these, but it is utterly inadequate to cope with the large numbers requiring treatment.

The proposals necessitating the extension include increased accommodation as follows: General medical beds, 177, to increase to 251; general surgical beds, 191, to increase to 401.

The special departments are as follows:

| | | | |
|---|---|---|---|
| Gynæcological ... ... | 12, | increased to | 36 |
| Maternity (new dept.) ... | — | | 27 |
| Aural ... ... ... ... | 17 | ,, | 27 |
| Ophthalmic ... ... ... | 18 | ,, | 27 |
| Orthopædic (new dept.) ... | — | | 20 |
| Skin ... ... ... ... | 15 | ,, | 15 |
| Venereal (new dept.) ... | — | ,, | 28 |
| Noisy cases and isolation (new dept.) ... ... | — | | 12 |
| | 430 | ,, | 844 |

The infirmary managers have not had any direct representation from the military authorities with regard to the orthopædic department, but it is, of course, well known to them that an orthopædic department with proper provision for massage and electrical treatment is a necessity now, and this necessity is increasing every month. Already there are a number of discharged soldiers which the managers are not able to deal with on the scale required. The local War Pensions Committees are making application for treatment, and there is money available for the payment of such treatment.

The present nurses' home accommodated about 120 nurses. It is proposed to add further accommodation to bring the number up to 300. A training-school for nurses will also be provided where probationers will undergo a three months' preliminary education before being sent to the wards. It is proposed to erect a private nursing institution from which nurses can be supplied for private cases outside, and to which institution they will return for further training from time to time in the infirmary wards.

The Town Moor and Parks Committee, after receiving a deputation from the infirmary, agreed to recommend the Council to grant the necessary land subject to the following conditions:

1. That the House Committee undertake to enclose the site with a wall and railing of the same design and construction as that enclosing the existing infirmary site.

2. That the House Committee also undertake to lay out, plant with trees, and maintain as a plantation the land coloured round with green upon the plan (containing an area of 1¼ acres or thereabouts) to the satisfaction of the Town Moor and Parks Committee, the Corporation reserving the right to maintain in its present position the existing sewer under that portion of the site, together with rights of access for reconstruction (including enlargement) and repair and maintenance of the said sewer.

3. That the House Committee undertake to construct a footpath of tar macadam or other approved material, 10 feet in width, along the north-west side of the site to the satisfaction of the Town Moor and Parks Committee.

4. That the House Committee undertake to pay (i.) the cost of any proceedings which may become necessary in respect of the closing of the public footpath or footpaths across the site, the Council on their part undertaking to use their best endeavours to obtain the closing of the same, and (ii.) the cost of the promotion of a Bill by the Corporation and the Stewards' Committee of the Freemen for the purpose of empowering the Corporation and the Freemen and the House Committee to carry the proposals into effect, and of vesting the site in the trustees of the infirmary as a site for a hospital or infirmary for the sick and maimed poor of the city and of the adjoining counties of Northumberland and Durham.

Messrs. Newcombe and Newcombe, Eldon Square, Newcastle, are at present engaged on the official plans. The only plans prepared up to date are merely diagrammatic to show the area required. The Bill for Parliament will not be ready until October, and at the present moment a portion of the ground is by mutual arrangement being used by the military for tent hospitals. These will be taken down by October.

